# Cryo-Electron Tomography Reveals the Complex Ultrastructural Organization of Multicellular Filamentous *Chloroflexota* (*Chloroflexi*) Bacteria

**DOI:** 10.3389/fmicb.2020.01373

**Published:** 2020-06-26

**Authors:** Vasil A. Gaisin, Romain Kooger, Denis S. Grouzdev, Vladimir M. Gorlenko, Martin Pilhofer

**Affiliations:** ^1^Research Center of Biotechnology of the Russian Academy of Sciences, Moscow, Russia; ^2^Algatech, Institute of Microbiology of the Czech Academy of Sciences, Třeboň, Czechia; ^3^Institute of Molecular Biology & Biophysics, Eidgenössische Technische Hochschule Zürich, Zurich, Switzerland

**Keywords:** ultrastructural organization, *Chloroflexi*, *Chloroflexota*, cryo-electron tomography, filamentous bacteria, multicellular bacteria, cell envelope, intracellular organelles

## Abstract

The cell biology of *Chloroflexota* is poorly studied. We applied cryo-focused ion beam milling and cryo-electron tomography to study the ultrastructural organization of thermophilic *Roseiflexus castenholzii* and *Chloroflexus aggregans*, and mesophilic “*Ca*. Viridilinea mediisalina.” These species represent the three main lineages within a group of multicellular filamentous anoxygenic phototrophic *Chloroflexota* bacteria belonging to the *Chloroflexales* order. We found surprising structural complexity in the *Chloroflexales*. As with filamentous cyanobacteria, cells of *C. aggregans* and “*Ca*. Viridilinea mediisalina” share the outer membrane-like layers of their intricate multilayer cell envelope. Additionally, cells of *R. castenholzii* and “*Ca*. Viridilinea mediisalina” are connected by septal channels that resemble cyanobacterial septal junctions. All three strains possess long pili anchored close to cell-to-cell junctions, a morphological feature comparable to that observed in cyanobacteria. The cytoplasm of the *Chloroflexales* bacteria is crowded with intracellular organelles such as different types of storage granules, membrane vesicles, chlorosomes, gas vesicles, chemoreceptor-like arrays, and cytoplasmic filaments. We observed a higher level of complexity in the mesophilic strain compared to the thermophilic strains with regards to the composition of intracellular bodies and the organization of the cell envelope. The ultrastructural details that we describe in these *Chloroflexales* bacteria will motivate further cell biological studies, given that the function and evolution of the many discovered morphological traits remain enigmatic in this diverse and widespread bacterial group.

## Introduction

There are only two groups of filamentous multicellular phototrophic bacteria: a polyphyletic group of filamentous cyanobacteria and green non-sulfur bacteria, which belong to the *Cyanobacteria* and *Chloroflexota* (*Chloroflexi*) phyla, respectively. Despite green non-sulfur bacteria and filamentous cyanobacteria being phylogenetically distant lineages that have many differences in their biology, their phenotypes often share many significant similarities that are adapted to specific ecological niches. Their multicellular filaments often form a dense “fabric” of cyanobacterial mats or biofilms (Bauld and Brock, 1973; Doemel and Brock, 1977; Ley et al., 2006; Gaisin et al., 2015). In addition to their the multicellular filamentous morphology, the cells contain (bacterio)chlorophylls that are essential to a phototrophic lifestyle. In addition, they employ comparable surface-dependent “gliding” motility to migrate through a mat or form aggregates (Richardson and Castenholz, 1987; Hanada, 2014). Thus, filamentous cyanobacteria and green non-sulfur bacteria possibly present an example of convergent evolution that has led to resembling phenotypes in phylogenetically distant lineages. Therefore, we postulated that studying the differences and similarities between these two groups would help understand the evolution of the morphological traits associated with multicellular phototrophic bacteria. To be able to compare the morphologies between these two phyla, their ultrastructural traits first need to be characterized. However, in contrast to cyanobacteria, the cell architecture of *Chloroflexota* bacteria is poorly understood.

Green non-sulfur bacteria is an outdated term used to define phototrophic species of the *Chloroflexota* phylum. The majority of phototrophic *Chloroflexota* bacteria belong to the *Chloroflexales* order (Grouzdev et al., 2018; Thiel et al., 2018). All *Chloroflexales* bacteria display a branchless filamentous multicellular morphology (Hanada, 2014). Their cells are colored because they contain bacteriochlorophylls and carotenoids. In all but one case, no noticeable ultrastructural differences have been reported between cells of an individual multicellular filament, leaving it unclear whether cell specialization occurs, as in the case of many cyanobacterial species. *Oscillochloris chrysea*, in which the terminal cells have a morphology distinct from the rest of the filament, is the only exception (Gorlenko and Pivovarova, 1977; Garrity et al., 2001). In contrast, many filamentous cyanobacteria possess specialized cells, such as vegetative cells, heterocysts and akinetes. In general, *Chloroflexales* bacteria seem to display a simpler organization than cyanobacteria, although this view might originate from the techniques that have been applied for their imaging.

Progress in cryo-electron microscopy has enabled new advances in the exploration of cyanobacterial cell biology, leading to the discovery of intricate macromolecular details (Dai et al., 2018; Rast et al., 2019; Weiss et al., 2019). As of today, ultrastructural data on *Chloroflexales* bacteria are relatively scarce. Indeed, the discovery and description of ultrastructural traits in *Chloroflexales* bacteria have been confined to those readily detected by “conventional” electron microscopy, such as negative stain transmission electron microscopy (TEM) (Pierson and Castenholz, 1974; Gorlenko and Pivovarova, 1977; Keppen et al., 1994; Hanada et al., 1995, 2002; Gaisin et al., 2019a, b). Although very insightful, this method usually does not expose the finer ultrastructural complexity of biological specimens.

Cryo-electron tomography (cryo-ET) is an electron microscopy technique that allows macromolecular structures to be studied at a resolution of approximately 4 nm and in 3D, while also preserving the sample in a near-native state (Pilhofer et al., 2010; Beck and Baumeister, 2016). Cryo-ET is limited to the imaging of thin samples (<700 nm). Therefore, imaging thicker bacteria has been dependent on the advances in sample thinning techniques such as cryo-focused ion beam (cryo-FIB) milling (Marko et al., 2007). Here, we used cryo-ET and cryo-FIB milling followed by cryo-ET to study the ultrastructural organization of the thermophilic *Roseiflexus castenholzii*, thermophilic *Chloroflexus aggregans*, and the recently described mesophilic bacterium “*Ca*. Viridilinea mediisalina” (Hanada et al., 1995, 2002; Gaisin et al., 2019a). We chose these species because each represents one of the three main lineages within the *Chloroflexales* order: bacteriochlorophyll *a*-containing *Roseiflexus*-related members, bacteriochlorophyll *a* and *c*-containing *Chloroflexus*-related members, and a group of mesophilic bacteriochlorophyll *a*, *c* and *d*-containing members with gas vesicles. The results presented here allow for a deeper understanding of the cell biology of *Chloroflexales*, as well as *Chloroflexota* bacteria in general. Particularly, these data provide new information to the debate surrounding the organization of the cell envelope in *Chloroflexota* (Sutcliffe, 2011; Cavalier-Smith and Chao, 2020), the mechanism of their motility and adherence (Fukushima et al., 2016; Fukushima, 2016), and finally on the multicellular organization of the phototrophic bacteria.

## Materials and Methods

### Bacterial Cultures

Culture of *R. castenholzii* DSM 13941 was grown in liquid medium consisting of the following components (g l^–1^): 0.5 KH_2_PO_4_, 0.5 NH_4_Cl, 0.3 MgCl_2_⋅6H_2_O, 0.5 KCl, 0.5 NaCl, 0.5 Na_2_SO_4_, 0.2 Na_2_S⋅9H_2_O, 0.3 NaHCO_3_, 2.0 yeast extract, 3.0 HEPES. The medium was completed by Wolfe’s vitamin (10 ml l^–1^) and trace element (10 ml l^–1^) solutions from ATCC. The pH of the medium was adjusted to 7.7. The culture was maintained in a glass tube with screw caps and rubber septa at a temperature of 50.5°C under constant light (incandescent light bulb).

Culture of *C. aggregans* DSM 9485 was grown in liquid medium consisting of the following components (g l^–1^): 0.5 KH_2_PO_4_, 0.5 NH_4_Cl, 0.3 MgCl_2_⋅6H_2_O, 0.5 KCl, 0.5 NaCl, 0.5 Na_2_SO_4_, 0.5 Na_2_S⋅9H_2_O, 0.3 NaHCO_3_, 1.5 yeast extract, 3.0 HEPES. The medium was complemented with the 1000x trace element solution (1 ml l^–1^) and iron (III) citrate (0.006 g l^–1^) as described for BG-11 medium (Rippka et al., 1979). The pH of the medium was adjusted to 7.7. The culture was maintained in a glass vial with screw caps and rubber septa at a temperature of 55.0°C under constant light (incandescent light bulb).

Culture of “*Ca*. Viridilinea mediisalina” Kir15-3F was grown in liquid medium consisting of the following components (g l^–1^): 0.2 KH_2_PO_4_, 0.2 NH_4_Cl, 0.2 MgCl_2_⋅6H_2_O, 0.3 KCl, 25.0 NaCl, 0.3 Na_2_SO_4_, 0.3 Na_2_S_2_O_3_ 0.7 Na_2_S⋅9H_2_O, 0.01 CaCl_2_⋅2H_2_O, 0.6 NaHCO_3_, 0.1 sodium acetate. The medium was supplied by 100x MEM vitamins 10 ml l^–1^, the 1000x trace element solution (1 ml l^–1^) and iron (III) citrate (0.006 g l^–1^) as described for BG-11 medium (Rippka et al., 1979). The pH of the medium was adjusted to 9.26. The culture was maintained in glass vials with screw caps and rubber septa at a temperature of 32°C under constant light [fluorescent tube (JBL SOLAR COLOR T8)].

For all strains, the condition of the cultures (motility, cell integrity, general appearance) was checked under a light microscope Zeiss Axiovert 200M, with 10X–63X NA 1.4 DIC objectives and images were collected on a Hamamatsu ORCA-ER CR4742-95 (1.3 k × 1 k, 6.45 μm × 6.45 μm).

### Preparation of Frozen-Hydrated Specimens

Plunge freezing was performed as described by Weiss et al. (2017). 27 μL bacterial samples were mixed with 5 μL protein A – 10 nm gold conjugate (Cytodiagnostics Inc.). Samples for cryo-focused ion beam (cryo-FIB) milling were frozen without gold fiducial markers. A 3.5 μL droplet of the sample was applied to a carbon-coated EM copper grid (R2/1 or R2/2, Quantifoil) that had been previously glow-discharged for 30–45 s at -25 mA using a Pelco easiGlowTM (Ted Pella, Inc.). The grids were plunge-frozen in liquid ethane-propane (37%/63%) using a Mark IV Vitrobot (Thermo Fisher Scientific). The forceps were mounted in the Vitrobot (22.5°C, humidity 100%) and the grids were blotted for 4–7 s from both sides or only from the backside by installing a Teflon sheet (instead of a filter paper) on the front blotting pad. Grids were stored in liquid nitrogen.

### Cryo-Focused Ion Beam Milling

Cryo-FIB milling was used to prepare samples of plunge-frozen cells of *R. castenholzii* and “*Ca*. Viridilinea mediisalina” that could then be imaged by cryo-ET. “*Ca*. Viridilinea mediisalina” and *R. castenholzii* were covered by an electron-dense extracellular matrix that dramatically decreased the contrast of the images. For this reason, their cells were pre-processed by cryo-FIB, a sample-thinning technique that essentially does not affect the native preservation of the sample (Marko et al., 2007). Tomograms of *C. aggregans* were collected on the bacterial cells directly after plunge-freezing, that is, without applying any specific preparative steps. Our cryo-FIB milling workflow has been detailed previously (Medeiros et al., 2018). Applied conditions for the cryo-FIB milling were the same as described recently (Rapisarda et al., 2019). Up to 7 lamellae were milled per grid. The grids were unloaded and stored in liquid nitrogen.

### Cryo-Electron Tomography

Cryo-FIB-processed *R. castenholzii* and “*Ca*. Viridilinea mediisalina” cells and unprocessed *C. aggregans* frozen cells were imaged by cryo-ET. Images were recorded on a Titan Krios TEM (Thermo Fisher Scientific) equipped with a Quantum LS imaging filter and K2 Summit (Gatan). The microscope was operated at 300kV and the imaging filters with a 30 eV slit width. The pixel size at the specimen level was 4.34 Å. Tilt series covered an angular range from −60° to +60° with 2° increments and −10 to −8 μm defocus. The total dose of a tilt series was 110–140 e-/Å^2^. Tilt series and 2D projection images were acquired automatically using SerialEM (Mastronarde, 2005). Three-dimensional reconstructions and segmentations were generated using the IMOD program suite (Kremer et al., 1996). All tomographic slices shown were from tomograms that had been previously deconvolved with a Wiener-like filter (Tegunov and Cramer, 2019).

### Sub-Tomogram Averaging for S-Layer

The tomogram used for sub-tomogram averaging was not corrected for the contrast transfer function, as it was not deemed necessary for the targeted final resolution. Individual particles were identified visually and manually modeled with open contours in 3dmod (Kremer et al., 1996). The manual particle picking and first round of sub-tomogram averaging were performed with the PEET (Particle Estimation for Electron Tomography) software package on tomograms that were binned by 4 (1k reconstructions). Model points, the initial motive list, and the particle rotation axes were generated using the stalkInit program from the PEET package (Nicastro, 2006). This approach allowed the definition of each structure’s longitudinal axis as the particle y-axis. The individual particles (*n* = 1498) were extracted from a tomogram of “*Ca*. Viridilinea mediisalina” using PEET. The final average had a box size of 48 pixels in × and z, and 68 pixels in y for the final step on data binned by 2 (“2k reconstruction,” final pixel size 9.88 Å). A single particle was chosen as a first reference. A cylindrical mask was applied. Missing wedge compensation was activated. An initial average was performed on data that were binned by 4 (“1k reconstruction”), and the final motive lists were then translated and used to perform a new round of subtomogram averaging on tomograms that were binned by 2 (2k reconstructions). C6 symmetry was imposed based on the evident hexagonal arrangement of the S-layer and of the non-symmetrized average. The Fourier shell correlation curves were calculated in PEET to estimate resolution. The model for visualization was generated from the averages and overlaid in tomographic slices in UCSF Chimera.

### Data Availability

A representative tomogram for each of the three strains has been deposited to the EMDB: EMD-10808 (“*Ca*. Viridilinea mediisalina” Kir15-3F), EMD-10806 (*C. aggregans* DSM 9485), EMD-10807 (*R. castenholzii* DSM 13941). The S-layer average has been deposited to the EMDB under number EMD-10804.

### Comparative Genomic and Phylogenetic Analysis

Search using the protein family corresponding to cell wall structure and intracellular granules biosynthesis was performed in Annotree (AnnoTree v1.1.0; GTDB Bacteria Release 03-RS86) (Mendler et al., 2019). Phylogenomic analysis of *Chloroflexales* was conducted using a concatenated alignment of 120 single-copy phylogenetic marker genes obtained using the software GTDB-Tk version 0.3.3 (Chaumeil et al., 2019). Maximum likelihood trees were calculated using IQ-Tree (Nguyen et al., 2015) using model LG+F+I+G4 recommended by ModelFinder (Kalyaanamoorthy et al., 2017) and branch support was estimated using UFBoot2 (Hoang et al., 2018). The list of genomes included in the analysis is presented in Supplementary Table S1. The evolution of genes encoding proteins for the biogenesis of intracellular bodies was studied by reconciling protein trees built from protein-by-protein alignments of GlpX, Ppk1, PhaC, BchK, and GvpN with the species tree under the duplication-transfer-loss parsimony algorithm implemented in Notung 2.9 software (Stolzer et al., 2012). The algorithm captures gene duplication, transfer and loss driving tree incongruence and infers all optimal solutions to finally report the complete and temporally feasible event histories giving the data.

## Results and Discussion

### Cell Envelope Architecture

**FIGURE 1:**
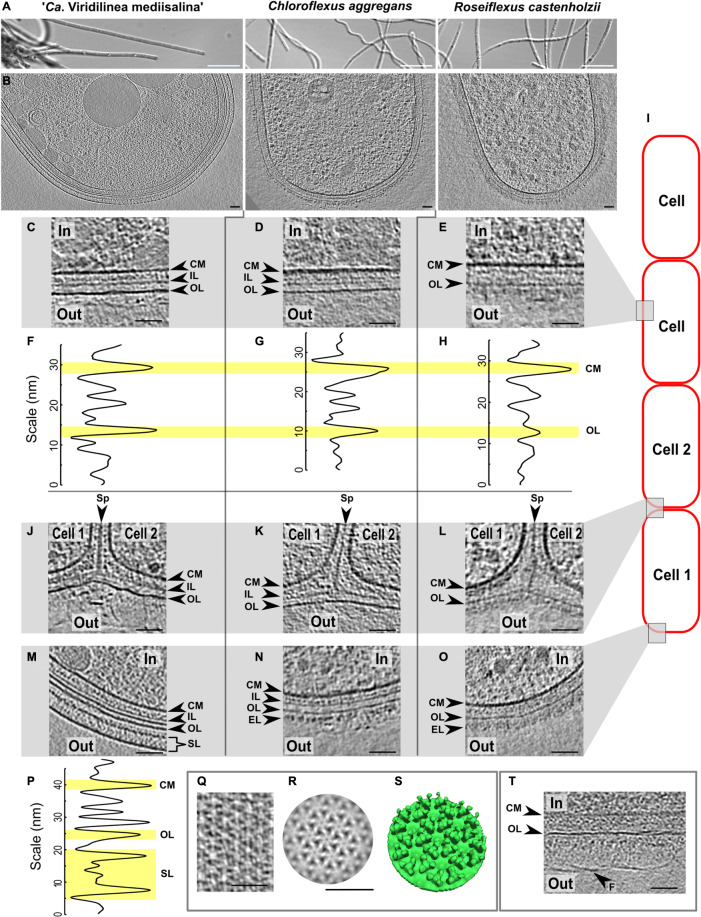
Comparative cryo-ET of the cell envelope of the *Chloroflexales* bacteria. Morphology of the strains in a light microscope **(A)**. Slices through tomograms of a terminal cell in the multicellular filaments **(B)**. Slices through tomograms of a middle part of the cells in “*Ca*. Viridilinea mediisalina” **(C)**, *C. aggregans* **(D)**, *R. castenholzii* **(E)**. Density profile of the cell envelope on the middle part of the cells in “*Ca*. Viridilinea mediisalina” **(F)**, *C. aggregans* **(G)**, *R. castenholzii* **(H)**. Slices through tomograms of a cell-cell junctions in “*Ca*. Viridilinea mediisalina” **(J)**, *C. aggregans* **(K)**, *R. castenholzii* **(L)**. Slices through tomograms of an apex of the terminal cells in “*Ca*. Viridilinea mediisalina” **(M)**, *C. aggregans* **(N)**, *R. castenholzii* **(O)**. Density profile of the cell envelope in the apex of the “*Ca*. Viridilinea mediisalina” terminal cell **(P)**. S-layer on tomogram without cryo-FIB **(Q)**. Sub-tomogram averaging of the S-layer **(R)** and its isosurface **(S)**. Fibrillar layer on a middle part of the cells in “*Ca*. Viridilinea mediisalina” **(T)**. In, cytoplasm in a cell; out, extracellular space; CM, cytoplasmic membrane; EL, external layer; FL, fibrillar layer; F, fibrils; IL, intermediate layer; OL, outer layer; SL, S-layer; Sp, septum. Bars: 10 μm **(A)**, 50 nm **(B–T)**. Scheme of the bacterial multicellular filament **(I)**.

“*Ca*. Viridilinea mediisalina,” *C. aggregans*, and *R. castenholzii*, showed a typical filamentous multicellular morphology when observed under an optical microscope (Figure 1A). Representative slices through tomograms of cells at the end of a filament are shown in Figure 1B for each of the three strains. We reconstructed 34 tomograms of “*Ca*. Viridilinea mediisalina,” 24 tomograms of *C. aggregans*, and 31 tomograms of *R. castenholzii*.

The architecture of the multilayered cell envelope of *Chloroflexota* has been a source of debate for a long time. Among others, some points of contention have centered around some micrographs that arguably do not allow to undoubtedly determine discrete layers in the cell envelope (Cavalier-Smith, 2006; Sutcliffe, 2011; Cavalier-Smith and Chao, 2020). Our tomograms revealed that both “*Ca*. Viridilinea mediisalina” and *C. aggregans* showed an obvious diderm-like cell envelope, whereas the organization of the cell envelope was different in *R. castelholzii*. Due to this difference and owing to the lack of evidence from biochemical and genetic experiments, we decided to only explicitly define the cytoplasmic membrane, while assigning more neutral terms to the other layers of the cell envelope: intermediate layer and outer layer.

For “*Ca*. Viridilinea mediisalina” and *C. aggregans*, we clearly distinguished three layers: a cytoplasmic membrane, an intermediate layer and an outer layer (Figures 1C,D,F,G,J,K,M,N). For these two strains, the intermediate layer was characteristic of a peptidoglycan layer of a Gram-negative bacterium (Tocheva et al., 2011). The appearance of the outer layer in these two strains resembles that of a typical Gram-negative outer membrane. However, the outer layer in *C. aggregans* appeared slightly less electron-dense than the cytoplasmic membrane (Figures 1D,K,N). In contrast, for the third strain, *R*. *castelholzii*, we only identified two continuous densities at the cell envelope: a cytoplasmic membrane and an outer layer (Figures 1E,H,L,O). Remarkably, the outer layer of *R*. *castelholzii* was reminiscent of the intermediate layer in *C. aggregans* and “*Ca*. Viridilinea mediisalina”.

The similarity between the outer layer of *R*. *castelholzii* and the intermediate layer of “*Ca*. Viridilinea mediisalina” and *C. aggregans* was clearly visible in tomograms of a septum between cells. For all three strains, the tomograms showed that each cell was enclosed by its own cytoplasmic membrane (Figures 1J,K,L). In “*Ca*. Viridilinea mediisalina” and *C. aggregans*, the intermediate layer was observed as a continuous density that surrounded the cytoplasmic membrane and that fused to form a septum shared between neighboring cells, as typically observed for a peptidoglycan layer (Figures 1J,K). Lastly, in these same two strains, the outer layer appeared as a third and external continuous density that was shared by all cells in the filament and that was not connected to the septum made by the intermediate layer (Figures 1J,K). Such an outer membrane shared by all cells of a filament is also seen in filamentous multicellular cyanobacteria, which are classified as diderm bacteria (Hoiczyk and Baumeister, 1995; Nicolaisen et al., 2011). In contrast, in *R. castenholzii*, the outer layer branched out toward the septum, in a similar manner to the intermediate layer in “*Ca*. Viridilinea mediisalina” and *C. aggregans* (Figure 1L). Given that both the thickness and the organization at the septum of the outer layer of *R. castenholzii* resemble that of the peptidoglycan layer in “*Ca*. Viridilinea mediisalina” and *C. aggregans*, it can be hypothesized that the outer layer in *R. castenholzii* represents the peptidoglycan and that there is no outer membrane. However, this layer of *R. castenholzii* did not resemble the typically thick peptidoglycan layer of a Gram-positive bacterium (Beeby et al., 2013; Tocheva et al., 2013).

Although our cryo-ET data provides insightful indications, it does not allow us to reach conclusions regarding the architecture of the cell envelope in these three *Chloroflexus* strains. We found that the cell envelope of “*Ca*. Viridilinea mediisalina” and *C. aggregans* possessed a diderm-like architecture. However, the nature of the outer layer in these strains remains enigmatic. Neither biochemical nor bioinformatic studies could reveal the presence of a typical outer membrane in *Chlorolexales* bacteria. For instance, lipopolysaccharides (LPS) have not been detected in *C. aurantiacus* and *O*. *trichoides* (Knudsen et al., 1982; Meissner et al., 1988; Keppen et al., 2018). Moreover, crucial genes of the LPS biosynthesis pathway have not been found in *Chlorolexales* or in other *Chloroflexota* species (Cavalier-Smith, 2006; Sutcliffe, 2010; Antunes et al., 2016; Keppen et al., 2018). In addition, we also analyzed *Chloroflexota* genomes, including the newly described “*Ca*. Viridilinea mediisalina,” for the presence of genes of the LPS biosynthesis (Supplementary Table S1). We did not find crucial genes such as *lpxC, lpxH, lpxB, lpxK, kdtA, kdsA*, *kdsB, lolA, lolB, lolCE*, and *lolD* (Supplementary Table S1). Another signature of the diderm architecture is the Bam complex, which assembles a β-barrel in the outer membrane of Gram-negative bacteria (Hagan et al., 2011). We did not find any homologs of the *bamA* gene in genomes of the strains analyzed here (Supplementary Table S1), in line with previous reports (Sutcliffe, 2010). To conclude, we can reconcile these biochemical and bioinformatic data with our microscopy data by stating that “*Ca*. Viridilinea mediisalina” and *C. aggregans* possibly have an atypical outer membrane that lacks many crucial features of the outer membrane of Gram-negative bacteria.

We were surprised to observe an exposed peptidoglycan layer and an apparent lack of outer membrane in tomograms of *R. castenholzii*, in particular since they belong to the same phylogenetic class as the two other strains. It is noteworthy that genomes of *R. castenholzii* and *Roseiflexus* sp. RS-1 do not possess *glgE, glgB, treS, treY treZ* genes for the biosynthesis of the branched α-glucan (Supplementary Table S1), which has been described in *O. trichoides* (Keppen et al., 2018). These genes are present in “*Ca*. Viridilinea mediisalina” and *C. aggregans*, as well as in other *Viridilinea*-related and other closely related *Chloroflexus* species (Supplementary Table S1). Thus, the strains with the outer membrane-like layer have a different peptidoglycan than *R. castenholzii*. Further studies are needed to decipher the characteristics of the peptidoglycan and how they are related to the architecture of the cell envelope in *Chloroflexales*.

### External Structures of the Cell Envelope

Additionally, all three *Chloroflexales* strains possessed an extra external density that covered the outer layer (Figures 1M–O). This density appeared as an amorphous extra layer ∼30–50 nm thick in *C. aggregans* and *R. castenholzii*, and was particularly dense in *R. castenholzii* (Figures 1N,O). In “*Ca*. Viridilinea mediisalina,” however, two other types of structures covered the outer layer. The curved apex of terminal cells was often capped by a crystalline mesh (Supplementary Figure S1). Side views of this structure revealed that it is formed of two planar densities 24 nm apart, 15 nm away from the outer layer (Figures 1M,P). We performed subtomogram averaging to obtain more structural information for repetitive elements of this layer (Supplementary Figure S1D). Given that a characteristic hexameric lattice was seen in top views, we provisionally describe it as an S-layer (Figures 1Q,R). Such a hexameric lattice of the S-layer has been for example recently described using cryo-ET and X-ray crystallography (Bharat et al., 2017). The average revealed that the vertices of the hexameric units were formed by trimeric pins (Figure 1S). The S-layer only formed at the apices. Elsewhere on the multicellular filament of “*Ca*. Viridilinea mediisalina,” the surface of the outer layer was covered by a dense fibrillar layer, which meshed into a planar density at ∼72 nm from the outer layer (Figure 1T and Supplementary Figures S1A,B).

This specific subcellular localization of the S-layer in “*Ca*. Viridilinea mediisalina” was surprising. Usually, S-layers completely cover the surface of a bacterial cell (Sleytr et al., 2014). Some multicellular filamentous cyanobacteria possess both an S-layer and a fibrillar outer layer (Hoiczyk and Baumeister, 1995; Hoiczyk, 1998). However, in these cyanobacteria, both layers coexist, and the fibrillar layer covers the S-layer.

In conclusion, cryo-ET showed that “*Ca*. Viridilinea mediisalina” has a more complex cell envelope than the thermophilic *C. aggregans* and *R. castenholzii*, which both lacked an S-layer. We assume that the specific localization of the S-layer hints at a particular function of the terminal cell in multicellular filaments.

### Cell-Cell Connections

**FIGURE 2:**
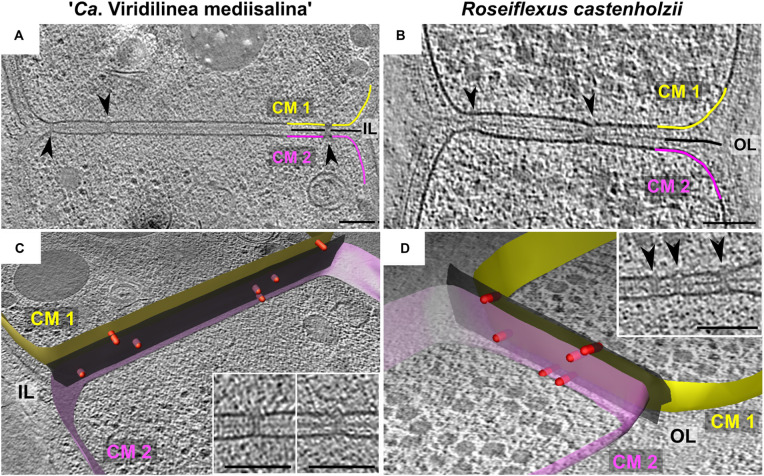
Slices through tomograms of septa between two “*Ca*. Viridilinea mediisalina” cells **(A)** and two *R. castenholzii* cells **(B)**. Segmentation of cell envelope and septal channels from tomograms of “*Ca*. Viridilinea mediisalina” **(C)** and *R. castenholzii* **(D)**: yellow (CM1) and pink surface (CM2) – cytoplasmic membranes of the two neighboring cells, black surface (Ol and IL) – outer and intermediate layer, red rods – the septal channels. Insertions in **(C,D)** show representative view of the septal channels. Arrows point the septal junctions. **(B,D)** show different tomograms of *R. castenholzii*. Bars: 100 nm.

We found that *R. castenholzii* and “*Ca*. Viridilinea mediisalina” had septal channels connecting neighboring cells across their shared septum (Figure 2), similar to the septal junctions in multicellular filamentous heterocyst-forming cyanobacteria (Merino-Puerto et al., 2010; Weiss et al., 2019). Clear evidence for the channels was absent in *C. aggregans*. Interestingly, we did not find homologs of the cyanobacterial genes responsible for the biogenesis of septal junctions in *Chloroflexota* genomes (*sepJ*, *fraC*, and *fraD* genes). In the absence of homologs, we propose the name septal channels to be adopted for these structures. The septal channels displayed a straight passage through the cell wall that created an indentation at the cytoplasmic membrane of both cells (Figures 2A,B). In some detailed examples, a hat-shaped cap covered the channel at the inner side of the cytoplasmic membrane (Figures 2C,D insertions). Cap-like structures have been observed on cyanobacterial septal junctions, where they are responsible for closing the channels in response to stress factors (Weiss et al., 2019). The existence of septal channels in *Chloroflexales* suggests that they are also capable of fast intercellular exchange of metabolites, similar to multicellular filamentous cyanobacteria. This hypothesis is interesting considering that fast intercellular exchange of metabolites is a feature of filamentous cyanobacteria that form metabolically specialized cells, such as, heterocyst (Mullineaux et al., 2008). However, such a metabolic cell specialization has not been reported for *Chloroflexales* bacteria.

### Pili and Receptor Array

**FIGURE 3:**
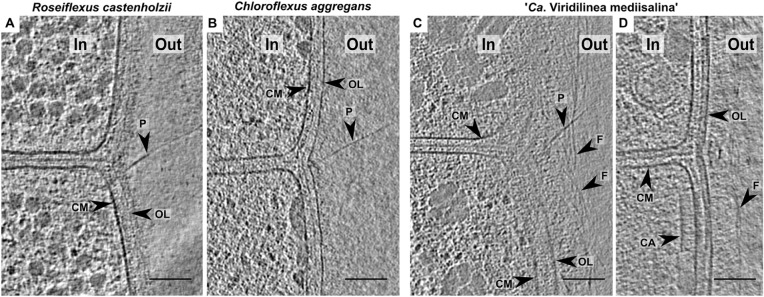
Slices through tomograms of the cells in *R. castenholzii* **(A)**, *C. aggregans* **(B)**, “*Ca*. Viridilinea mediisalina” **(C,D)**. In, cytoplasm in a cell; out, extracellular space; CM, cytoplasmic membrane; CA, chemoreceptor-like array; EL, external layer; F, fibrils; OL, outer layer; P, pilus. Bars: 100 nm.

*Chloroflexales* bacteria also possess extracellular appendages such as pili. Long pili have been previously reported in *C. aggregans* and *C. islandicus* (Fukushima, 2016; Gaisin et al., 2017). Here, we show that they were also present in *R. castenholzii* and “*Ca*. Viridilinea mediisalina.” The pili were anchored near the septa in the multicellular filaments (Figures 3A–C), similar to pilus localization in filamentous cyanobacteria (Khayatan et al., 2015). It was shown that the cyanobacterial pili are responsible for “gliding” motility (Khayatan et al., 2015). *Chloroflexales* strains are also capable of surface-dependent “gliding” motility, as well as active formation of aggregates (Hanada et al., 1995, 2002; Gaisin et al., 2019a). Therefore, we propose that the pili could mediate motility in *Chloroflexales* bacteria.

In cyanobacteria, motility is associated with type IV pili (Khayatan et al., 2015). However, the genomes of “*Ca*. Viridilinea mediisalina,” *C. aggregans*, and *R. castenholzii* only possess genes for Tad (tight adherence) pili biogenesis, such as *rcpC-tadZABC*, *tadEEG*, and *flp*. Indeed, most of the crucial genes for the type IV pilus system are absent, such as for example *pilA*, *pilC*, and *pilG*. Most bacteria rely on the type IV pilus system for pili-based motility (Burrows, 2012). In contrast, Tad pili have mainly been described as appendages that mediate surface adhesion (Tomich et al., 2007). This idea is supported by direct observations and the absence of a canonical pili retraction ATPase in a *tad* gene cluster (Burrows, 2012). Nevertheless, it has been shown that at a specific stage of the life cycle of *Caulobacter crescentus* the Tad pili can retract (Ellison et al., 2017). Based on these observations we assume that the Tad pili is the main candidate to the role of appendages driving surface-dependent motility in *Chloroflexales* bacteria.

An indication of active motile behavior in *Chloroflexales* was hinted at by the presence of structures resembling chemoreceptor arrays, which are usually associated with chemotaxis (Briegel et al., 2009). We found an array close to the septa in “*Ca*. Viridilinea mediisalina” (Figure 3D). The large array seemed to be anchored in the cytoplasmic membrane and extended ∼35 nm in the cytoplasm. It resembled typical bacterial chemoreceptor arrays (Briegel et al., 2009). Previously, Fukushima et al. suggested that *C. aggregans* was capable of aerotaxis (Fukushima, 2016), and a chemotaxis system in *Chloroflexales* has been predicted by genomic analysis (Wuichet and Zhulin, 2010). These previous findings and the images of chemoreceptor-like arrays presented here show that *Chloroflexales* bacteria are adapted to active translocation in their environment.

### Intracellular Granules

**FIGURE 4:**
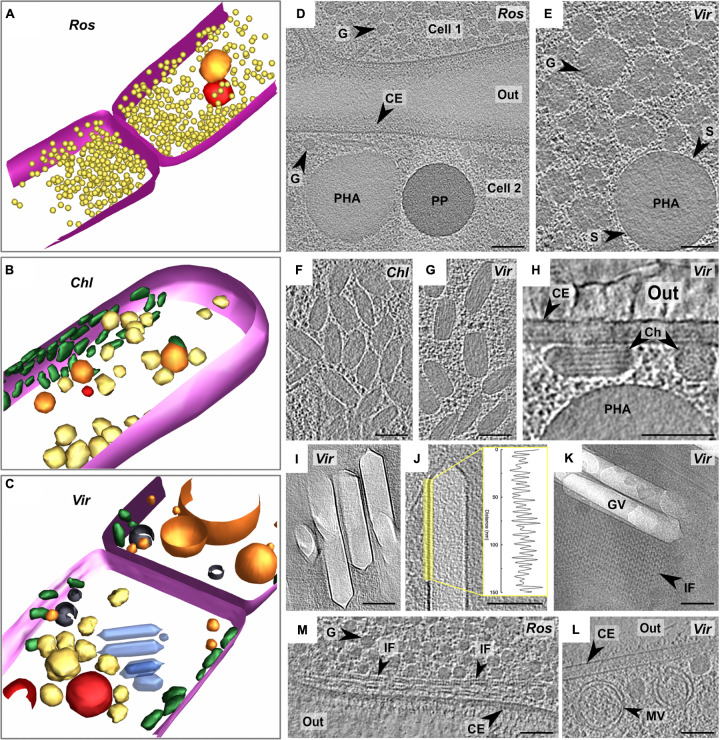
Segmentation of tomograms of *R. castenholzii* **(A)**, *C. aggregans* **(B)**, and “*Ca*. Viridilinea mediisalina” **(C)**: pink – cytoplasmic membrane, orange – polyhydroxyalkanoate granule, red – polyphosphate granule, yellow – third type of granule, green – chlorosomes, blue – gas vesicles, black – membrane vesicles. Marking of the strains on the images: *R. castenholzii* (Ros), *C. aggregans* (Chl), and “*Ca*. Viridilinea mediisalina” (Vir). Intracellular granules on slices through tomograms of *R. castenholzii* **(D)** and “*Ca*. Viridilinea mediisalina” **(E)**. Chlorosomes under CM on slices (top view) through tomograms of *C. aggregans* **(F)** and “*Ca*. Viridilinea mediisalina” **(G)**. Chlorosomes on slices (cross section view) through tomogram of “*Ca*. Viridilinea mediisalina” **(H)**. Gas vesicles on slices through tomogram of “*Ca*. Viridilinea mediisalina” **(I,J)** and density profile of the vesicles membrane. Gas vesicles and sheet of intracellular filaments on CryoEM image **(K)**. Intracellular filaments on slices through tomogram of *R. castenholzii* **(M)**. Intracellular vesicles on slices through tomogram of “*Ca*. Viridilinea mediisalina” **(L)**. In, cytoplasm in a cell; out, extracellular space; CE, cell envelope; Ch, chlorosome; G, third type of granule; GV, gas vesicle; IF, intracellular filaments; MV, membrane vesicle; PHA, polyhydroxyalkanoate granule; PP, polyphosphate granule; S, surface of polyhydroxyalkanoate granules. Bars: 100 nm.

Intracellular organelles in *Chloroflexales* have previously been identified using negative stain TEM, and we were able to compare published micrographs to our cryo-ET data (Pierson and Castenholz, 1974; Keppen et al., 1994; Hanada et al., 1995, 2002; van der Meer et al., 2010; Gaisin et al., 2017, 2019a,b). In our tomograms, we identified intracellular bodies such as storage granules, chlorosomes, and gas vesicles, which are shown in Figure 4. Additionally, we also identified genes that are responsible for the biogenesis of these organelles (Supplementary Table S1). The distribution of these genes is discussed in tandem with the description of the organelles.

We found storage granules in all three *Chloroflexales* bacteria (Figures 4A–E). We distinguished three types of storage granules. The first type consisted of relatively large spherical intracellular bodies (Supplementary Table S2, up to ∼700 nm in “*Ca*. Viridilinea mediisalina”) (Figures 4D,E). We identified these structures as putative polyhydroxyalkanoate granules since they typically show a distinctive electron-dense surface by cryo-ET (Beeby et al., 2012), similar to what we observed (Figure 4E). The ability to produce polyhydroxyalkanoate was supported by the presence of the *phaC* gene, which encodes a poly-3-hydroxyalkanoate polymerase (Steinbüchel et al., 1992), in the genomes across the *Chloroflexales* order (Supplementary Table S1). The second type of granules were more electron-dense spherical intracellular bodies (Supplementary Table S2, up to ∼280 nm in “*Ca*. Viridilinea mediisalina”) and were designated as polyphosphate granules (Figure 4D), in accordance with these being the most electron-dense granules in a bacterial cell in both conventional EM and cryoEM images (Racki et al., 2017). Genomes of all *Chloroflexales* also possess the *ppk1* gene of the polyphosphate kinase (Supplementary Table S1), which is involved in polyphosphate biogenesis (Achbergerová and Nahálka, 2011). We identified a third type of granule, which appeared as electron-dense as polyhydroxyalkanoate granules, but did not possess a distinct and regular surface. In “*Ca*. Viridilinea mediisalina” and in *C. aggregans*, these granules were globular, but less spherical and smaller (Supplementary Table S2, up to ∼150 nm in “*Ca*. Viridilinea mediisalina”) than polyhydroxyalkanoate granules (Figure 4E). Additionally, they showed innervations radiating inwards from the surface (Figure 4E). We hypothesize that these could be glycogen granules (Figures 4D,E), based on the presence of *glpX* gene encoding a fructose-1,6-bisphosphatase in the *Chloroflexales* genomes (Supplementary Table S1) and on published experimental data pointing toward storage of intracellular polyglucose in *C. aurantiacus* (Holo and Grace, 1987). In *R. castenholzii*, in addition to polyhydroxyalkanoate and polyphosphate granules, we observed smaller granules (Supplementary Table S2, 38 ± 4 nm, up to 45 nm) that were very uniform in size (Figure 4D). This third type of granules in *R. castenholzii* seemed different from the third type granules in *C. aggregans* and “*Ca*. Viridilinea mediisalina” because they had a more regular size and shape.

### Chlorosomes

Chlorosomes are light-harvesting organelles made principally of bacteriochlorophyll and providing an ecological advantage when competing for light energy (Orf and Blankenship, 2013). Their presence in *C. aggregans* and “*Ca*. Viridilinea mediisalina” has been detected previously by negative stain TEM (Hanada et al., 1995; Gaisin et al., 2019a). Biogenesis of the chlorosomes is predicted in many *Chloroflexales* species owing to the presence of a *bchK* gene (Supplementary Table S1), which encodes a bacteriochlorophyll *c* synthase (Frigaard et al., 2005). As expected from the absence of a *bchK* gene in the genome, chlorosomes were absent in *R. castenholzii* tomograms (Figure 4A). Chlorosomes in the plunge-frozen cells of *C. aggregans* and “*Ca*. Viridilinea mediisalina” were recognizable from their characteristic ellipsoidal shape (Figures 4F,G). “*Ca*. Viridilinea mediisalina” possessed on average wider chlorosomes than *C. aggregans* (Supplementary Table S2). The striations and tubular organization of the *Chloroflexales* chlorosomes were discernible in particularly detailed tomograms of “*Ca*. Viridilinea mediisalina” (Figure 4H). These striations resembled those reported in green sulfur bacteria (Oostergetel et al., 2007; Ganapathy et al., 2009). Our tomograms showed that chlorosomes were directly adjacent to the cytoplasmic membrane (Figure 4H). This cryo-ET data is in agreement with the previously reported direct contact of the *Chloroflexus* chlorosomes to the cytoplasmic membrane that has been shown using conventional TEM on freeze-fractured cells (Staehelin et al., 1978). The adjoining of chlorosomes to the cytoplasmic membrane in our tomograms contrasts with the situation observed on cryo-ET images of green sulfur bacteria, where chlorosomes have been shown to be separated from the cytoplasmic membrane by an 8 nm density that likely corresponds to the Fenna-Matthews-Olson (FMO) complex protein (Kudryashev et al., 2014). It is a well-known fact that the FMO complex is absent in *Chloroflexales*.

### Gas Vesicles

When imaged by negative stain TEM, gas vesicles have only been detected in mesophilic *Chloroflexales* represented by “*Ca*. Viridilinea mediisalina” (Gorlenko and Pivovarova, 1977; Keppen et al., 1994; Gorlenko et al., 2014; Gaisin et al., 2019a). Indeed, genes that are crucial for normal gas vesicle biogenesis, *gvpA* and *gvpN* (Tashiro et al., 2016), are only present in genomes of mesophilic species (Supplementary Table S1). In “*Ca*. Viridilinea mediisalina,” gas vesicles formed cylindrical compartments of variable length that were capped by conical ends with small rounded tips (Figures 4I–K and Supplementary Table S2). In clear examples, we could distinguish the “ribs” of Gvp proteins on the surface of gas vesicles (Figure 4J), which had a periodicity of 4.8 ± 0.5 nm, comparable to the 4.6 nm reported previously (McMaster et al., 1996). The gas vesicles in “*Ca*. Viridilinea mediisalina” were located near the septa, as in other mesophilic *Chloroflexales* (Gorlenko and Pivovarova, 1977; Keppen et al., 1994; Gorlenko et al., 2014). Despite the specific localization of gas vesicles at the septum, they did not appear to be connected with the septal cytoplasmic membrane of “*Ca*. Viridilinea mediisalina.”

### Intracellular Filaments

Interestingly, we also found arrays of filaments forming sheets that co-occurred and were aligned alongside gas vesicles and also next to burst vesicles (Figure 4K). These filaments were wavy with ∼18 nm between consecutive peaks. Neighboring filaments in a sheet were ∼18 nm apart. In addition, we found another type of filaments in *R. castenholzii* (Figure 4K). These filaments formed sheets near the septum also, as well as long spindles along the cytoplasmic membrane (Figure 4M).

### Intracellular Membrane Vesicles

Finally, we found single and double-membraned intracellular vesicles in all three analyzed strains (Figure 4L and Supplementary Figure S2). It appears that the single-membrane vesicles were formed through invagination of the cytoplasmic membrane, as seen for instance in *C. aggregans* (Supplementary Figure S2B). The membrane vesicles had a variety of sizes. We suggest that these intracellular membrane vesicles are a typical ultrastructural trait of *Chloroflexales* because they were present in all cells, regardless of the growing conditions, i.e., in both mesophilic and thermophilic species.

### Evolutionary Considerations

**FIGURE 5:**
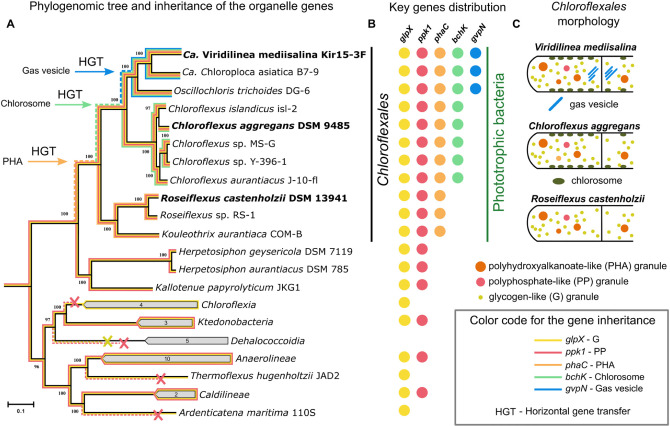
Maximum-likelihood phylogenetic tree (black line) inferred from concatenated alignment of 120 single-copy phylogenetic marker (GTDB dataset) genes **(A)**. List of genomes included in the phylogenetic analysis was presented in Supplementary Table S1. Distribution of g*lpX*, *ppk1*, *phaC*, *bchK*, and *gvpN* genes that encode the proteins for biogenesis of the intracellular bodies across *Chloroflexota* phylum **(B)**. Inheritance of the key genes is shown on the phylogenetic tree (colored lines). The dashed colored lines denote hypothetical inheritance from common ancestor of a clade. Schematic illustration of the main *Chloroflexales* morphotypes **(C)**.

The intracellular organelles like the storage granules, chlorosomes, and gas vesicles were easily traced across *Chloroflexales* using not only electron microscopy but comparative genomic analysis as well. We speculated about the evolution of *Chloroflexales* cell biological traits by analyzing the distribution of the described ultrastructural features and their relevant genes among *Chloroflexales* species. In this regard, we first reconstructed the phylogenetic relationship among *Chloroflexota* bacteria using 120 single-copy phylogenetic marker genes from the GTDB dataset (Figure 5A). Secondly, we reconstructed phylogenetic trees for the GlpX, Ppk1, PhaC, BchK, and GvpN proteins (Supplementary Figures S3–S7). The distribution of the corresponding genes among *Chloroflexota* genomes (Figure 5B and Supplementary Table S1) was already discussed above in the sections on the storage granules, chlorosomes, and gas vesicles. Thirdly, we performed gene-species tree reconciliation analysis based on the GTDB phylogenetic tree and trees of the GlpX, Ppk1, PhaC, BchK, and GvpN proteins using Notung software. The probability inferred from the reconciliation analysis allowed us to reconstruct the likely inheritance of the genes inferred to the intracellular organelles (Figure 5A). Lastly, we summarized the three main *Chloroflexales* morphotypes based on our results and previously published TEM data (Figure 5C).

We found that genes for polyphosphate and glycogen granules are not unique to phototrophic *Chloroflexota* bacteria. The *ppk1* gene was detected in the genomes of the classes *Ktedonobacteria*, *Anaerolineae*, *Caldilineae* (Figure 5B and Supplementary Table S1), and was inherited vertically from the common ancestor of these four classes according to the phylogenetic analysis (Supplementary Figure S3). The distribution of the *glpX* gene and result of the gene-species tree reconciliation analysis also indicated the vertical inheritance of this gene with independent losses in some groups, for example, *Dehalococcoidia* (Figure 5A). However, only *Chloroflexales* bacteria possess *phaC*, a crucial gene of the polyhydroxyalkanoate granules biogenesis (Figure 5 and Supplementary Table S1). In the phylogenetic tree, the sequences of the PhaC protein of *Chloroflexales* are clustered inside the clade formed by the bacteria of the *Firmicutes* phylum (Supplementary Figure S4). Thus, the most likely scenario is the acquisition of genes responsible for the synthesis of polyhydroxyalkanoates by *Chloroflexales* through horizontal transfer to their common ancestor from the ancient representative of the *Firmicutes* phylum (Figure 5A and Supplementary Figure S4). Horizontal gene transfer of the *phaC* gene to the *Chloroflexales* lineages was also inferred by the gene-species tree reconciliation analysis. The polyhydroxyalkanoate biogenesis and degradation in *Chloroflexales* bacteria are primarily dependent on diel cycling and coupled with their photomixotrophic metabolism (Klatt et al., 2013; Kim et al., 2015). Hence, this trait is important for the adaptation of the *Chloroflexales* bacteria to phototrophic metabolism.

Chlorosomes, another phototrophy-related trait, are absent in *Roseiflexus*-related bacteria, which form the basal phylogenetic lineage within the *Chloroflexales* clade (Figure 5). Genes for chlorosomes biogenesis were acquired by a common ancestor of the *Chloroflexus* and *Viridilinea*-related bacteria, according to our gene-species tree reconciliation analysis (Figure 5A) and the distribution of *csm* and *bchK* genes (Supplementary Table S1). The acquisition of this morphological trait increased the adaptability of *Chloroflexus* and *Viridilinea*-related bacteria to phototrophic ecological niches because chlorosomes provide dramatic additional light-absorption capabilities to cell (Orf and Blankenship, 2013).

The most complex intracellular composition was observed in the clade of mesophilic *Viridilinea*-related species (Figure 5C). The mesophilic *Viridilinea*-related bacteria have storage granules, chlorosomes, and additionally gas vesicles (Figure 5C). Gas vesicles have only been described in mesophilic *Chloroflexales* (Garrity et al., 2001; Hanada, 2014; Grouzdev et al., 2018). Genes for gas vesicles biogenesis were present only in genomes of *Viridilinea*-related species, i.e., they were absent in other *Chloroflexota* (Figure 5B and Supplementary Table S1). We assume that genes for gas vesicles biogenesis were acquired by a common ancestor of *Viridilinea*-related bacteria from an ancient representative of *Alphaproteobacteria* according to the results of phylogenetic (Supplementary Figure S5) and gene-species tree reconciliation analyses. Gas vesicles increase the buoyancy of cells in water, thereby allowing them to migrate through aquatic environments to reach the photic zone (Walsby, 1994). Hence, gas vesicles can favor distribution of mesophilic *Chloroflexales* bacteria in illuminated aquatic environments.

Phototrophy is the most significant synapomorphy acquired by a common ancestor of the current *Chloroflexales* bacteria (Shih et al., 2017; Ward et al., 2018). It has been postulated that a common ancestor of *Chloroflexales* was capable of aerobic respiration before the acquisition of photosynthetic genes (Shih et al., 2017; Ward et al., 2018). *Roseiflexus* and *Chloroflexus* species are also able to switch their anoxygenic phototrophic metabolism to aerobic respiration (Pierson and Castenholz, 1974; Hanada et al., 1995, 2002; Gaisin et al., 2017). In contrast, the *Viridilinea*-related species are obligate anoxygenic phototrophs and are obligate anaerobes (Keppen et al., 1994; Gorlenko et al., 2014; Gaisin et al., 2019a, b). We assume that the three acquisition events discussed above significantly contributed to the evolution of the mesophilic *Viridilinea*-related bacteria strictly specialized in phototrophy. Perhaps, the morphological complexity in this bacterial lineage evolved in conjunction with its fitness to a phototrophic lifestyle. As seen above, “*Ca*. Viridilinea mediisalina” has a more complex cell envelope than *C. aggregans* and *R. castenholzii* as it possesses for instance an S-layer. Therefore, mesophilic *Viridilinea*-related bacteria represent an interesting example among anoxygenic phototrophic bacteria of linages that evolved metabolic specialization along with a complex morphology.

## Conclusion

**FIGURE 6:**
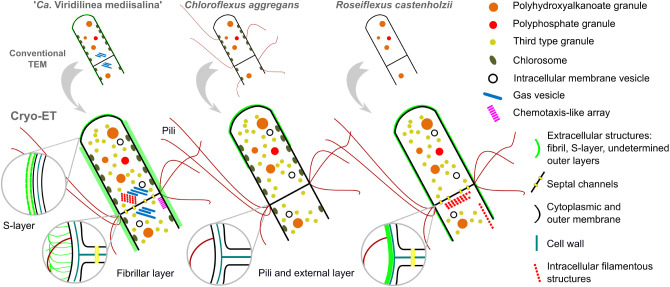
A new view on the cellular ultrastructural organization of *Chloroflexales* bacteria.

We have discovered a surprising degree of ultrastructural complexity in multicellular filamentous anoxygenic phototrophic bacteria that belong to the *Chloroflexales* order. Our cryo-ET data has revealed many interesting ultrastructural features of *Chloroflexales*, providing more detail on previously known structures, and also uncovering new ones (Figure 6). All cells have cytoplasms crowded with intracellular organelles. They contained organelles related to phototrophic metabolism (storage granules and chlorosomes), migration in an aquatic environment (gas vesicles and chemoreceptor-like arrays), and structures of unknown functions (intracellular membrane vesicles and filamentous sheets). In general, we found that mesophilic *Chloroflexales* displayed a particularly complex morphology. Moreover, the cell envelope of “*Ca*. Viridilinea mediisalina” possessed traits characteristic of multicellular microorganisms such as filamentous cyanobacteria, i.e., the distinct morphology of the terminal cells, the shared outer membrane and the septal channels. Also, it is remarkable that the pili are anchored close to the septum, similar to the anchoring pattern of pili in filamentous cyanobacteria. Both the organization of the cell envelope and the diversity of intracellular bodies indicate that mesophilic *Chloroflexales* display a particularly complex cell biology. In conclusion, we hope that the ultrastructural details that we described in the *Chloroflexales* bacteria will motivate further studies on the cell biology of these remarkable microorganisms, given that the function and evolution of many discovered morphological traits remain enigmatic in this diverse and widespread bacterial group.

## Data Availability Statement

The datasets presented in this study can be found in online repositories. The names of the repository/repositories and accession number(s) can be found in the article/Supplementary Material.

## Author Contributions

VAG and RK performed the experimental work, processed the cryo-ET data, and wrote the manuscript. DG performed the comparative genomic analyses and phylogenetic analyses. MP, VAG, and VMG coordinated the project. All authors discussed the results and approved the submitted version of the manuscript.

## Conflict of Interest

The authors declare that the research was conducted in the absence of any commercial or financial relationships that could be construed as a potential conflict of interest.
