# Supplementary material for: Cryo-Electron Tomography Reveals the Complex Ultrastructural Organization of Multicellular Filamentous Chloroflexota (Chloroflexi) Bacteria
Source: Front Microbiol. 2020 Jun 26;11:1373. doi: 10.3389/fmicb.2020.01373 (PMC7332563; doi:10.3389/fmicb.2020.01373)
Supplement: Supplementary file 2 [file Table_2.docx]

| **Organelle types** | **The organelle size (nm)** | | |
| --- | --- | --- | --- |
|  | ***R. castenholzii*** | ***C. aggregans*** | **‘*Ca*. V. mediisalina’** |
| *Polyhydroxyalkanoate granules* | 242 ± 119 | 165 ± 54 | 190 ± 171 |
| *Polyphosphate granules* | 153 ± 66* | 145 ± 66* | 153 ± 69* |
| *The third type granules* | 38 ± 4 | 81 ± 15 | 100 ± 24 |
| *Chlorosomes, width* | – | 34 ± 4 | 54 ± 10 |
| *length* | – | 126 ± 12 | 119 ± 22 |
| *Gas vesicles, width* | – | – | 74 ± 3* |
| *length* | – | – | 317 ± 98* |

Table S2. Size of the organelles as measured in the tomograms. Average ± standard deviation. (–) indicates that no structures were found in that strain. *N* ≥ 20, except for (*), where *N* ≥ 10.
