# Supplementary material for: Cryo-Electron Tomography Reveals the Complex Ultrastructural Organization of Multicellular Filamentous Chloroflexota (Chloroflexi) Bacteria
Source: Front Microbiol. 2020 Jun 26;11:1373. doi: 10.3389/fmicb.2020.01373 (PMC7332563; doi:10.3389/fmicb.2020.01373)
Supplement: Supplementary file 3 [file Data_Sheet_1.zip › Data Sheet 7.PDF]

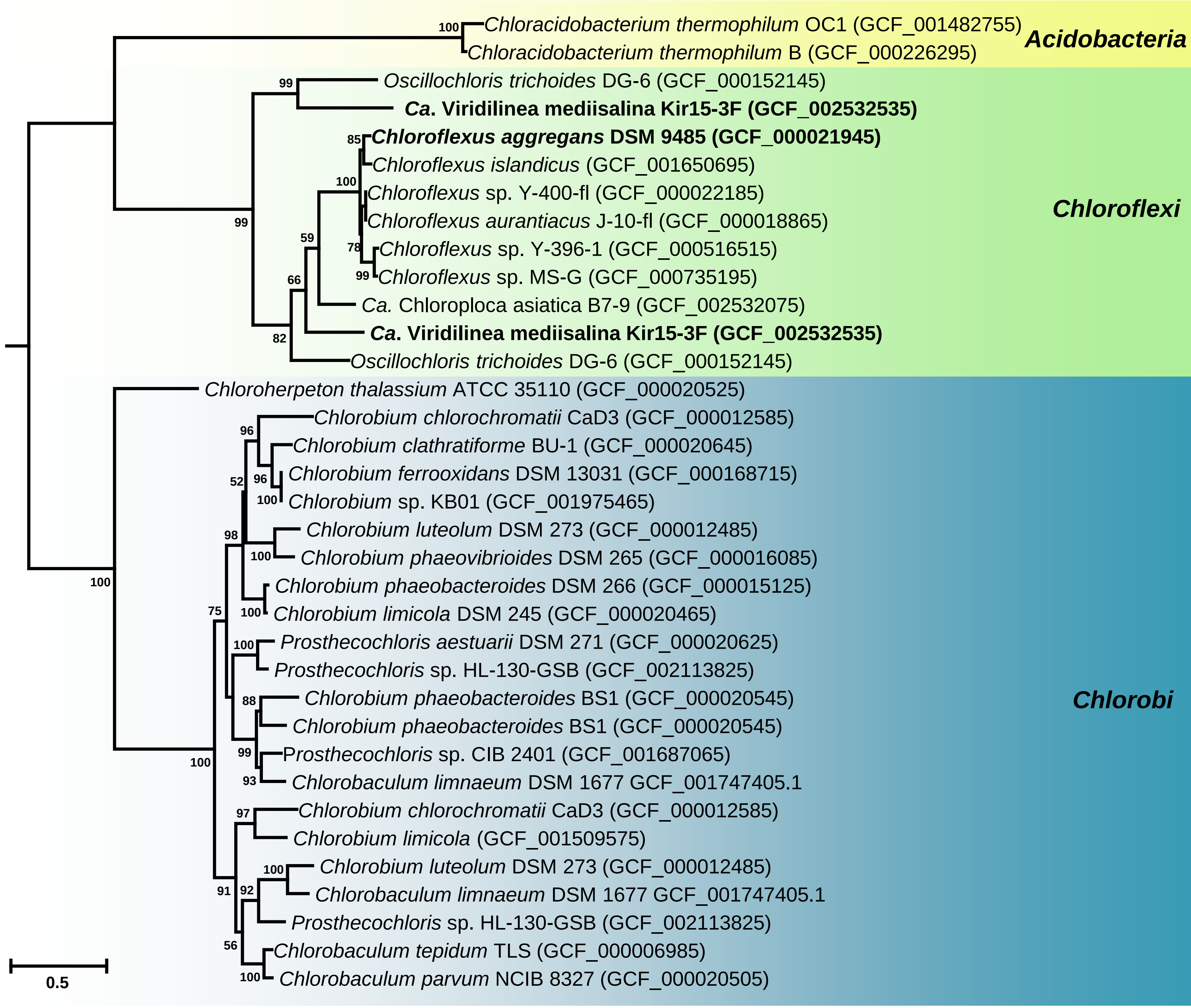

Supplementary Figure S7. Maximum likelihood phylogenetic tree of bacteriochlorophyll *c* synthase (BchK) amino acid sequences, based on a LG+F+I+G4 protein substitution model. Black numbers represent ultrafast bootstrap values (>50%) expressed in percent. The scale bar represents amino acid substitutions per site.
