# Supplementary material for: Cryo-Electron Tomography Reveals the Complex Ultrastructural Organization of Multicellular Filamentous Chloroflexota (Chloroflexi) Bacteria
Source: Front Microbiol. 2020 Jun 26;11:1373. doi: 10.3389/fmicb.2020.01373 (PMC7332563; doi:10.3389/fmicb.2020.01373)
Supplement: Supplementary file 3 [file Data_Sheet_1.zip › Supplementary Figure Captions.DOCX]

# Supplementary figure legend

**Supplementary Figure S1**. Top view of a surface on apex of a non-cryo-FIB-processed partially lysed terminal cell of '*Ca*. Viridilinea mediisalina' (A) and slices through the same tomograms on lower view in Z-axis (A2). Close-up views of filaments of the filamentous layer (B) and S-layer (C). FSC curves of the C6-symmetrized final average generated in the PEET software package (D). The n value in the top right box corresponds to the number of particles in each of the two equal-sized groups whose averages are being compared in the Fourier space. Bars: 100 (A and A2), 50 nm (B and C).

**Supplementary Figure S2.** Intracellular membrane vesicles (A). Invagination of cytoplasmic membrane and vesicle (B). CM – cytoplasmic membrane, IL – intermediate layer, OL – outer layer, PHA - polyhydroxyalkanoate granule, V – membrane vesicle. Bars: 100 nm.

**Supplementary Figure S3**. Maximum likelihood phylogenetic tree of poly P kinase 1 (PPK1) amino acid sequences, based on a LG+F+I+G4 protein substitution model. Black numbers represent ultrafast bootstrap values (>50%) expressed in percent. The scale bar represents amino acid substitutions per site.

**Supplementary Figure S4**. Maximum likelihood phylogenetic tree of polyhydroxyalkanoate synthase (PhaC) amino acid sequences, based on a LG+F+I+G4 protein substitution model. Black numbers represent ultrafast bootstrap values (>50%) expressed in percent. The scale bar represents amino acid substitutions per site.

**Supplementary Figure S5.** Maximum likelihood phylogenetic tree of gas vesicle protein GvpN amino acid sequences, based on a LG+I+G4 protein substitution model. Black numbers represent ultrafast bootstrap values (>50%) expressed in percent. The scale bar represents amino acid substitutions per site.

**Supplementary Figure S6**. Maximum likelihood phylogenetic tree of fructose-1,6-bisphosphatase (GlpX) amino acid sequences, based on a LG+F+I+G4 protein substitution model. Black numbers represent ultrafast bootstrap values (>50%) expressed in percent. The scale bar represents amino acid substitutions per site.

**Supplementary Figure S7**. Maximum likelihood phylogenetic tree of bacteriochlorophyll *c* synthase (BchK) amino acid sequences, based on a LG+F+I+G4 protein substitution model. Black numbers represent ultrafast bootstrap values (>50%) expressed in percent. The scale bar represents amino acid substitutions per site.
