# Supplementary figures and images for: Cryo-Electron Tomography Reveals the Complex Ultrastructural Organization of Multicellular Filamentous Chloroflexota (Chloroflexi) Bacteria
Source: Front Microbiol. 2020 Jun 26;11:1373. doi: 10.3389/fmicb.2020.01373 (PMC7332563; doi:10.3389/fmicb.2020.01373)

# Supplementary Figure S1

## A '*Ca. Viridilinea mediisalina*'

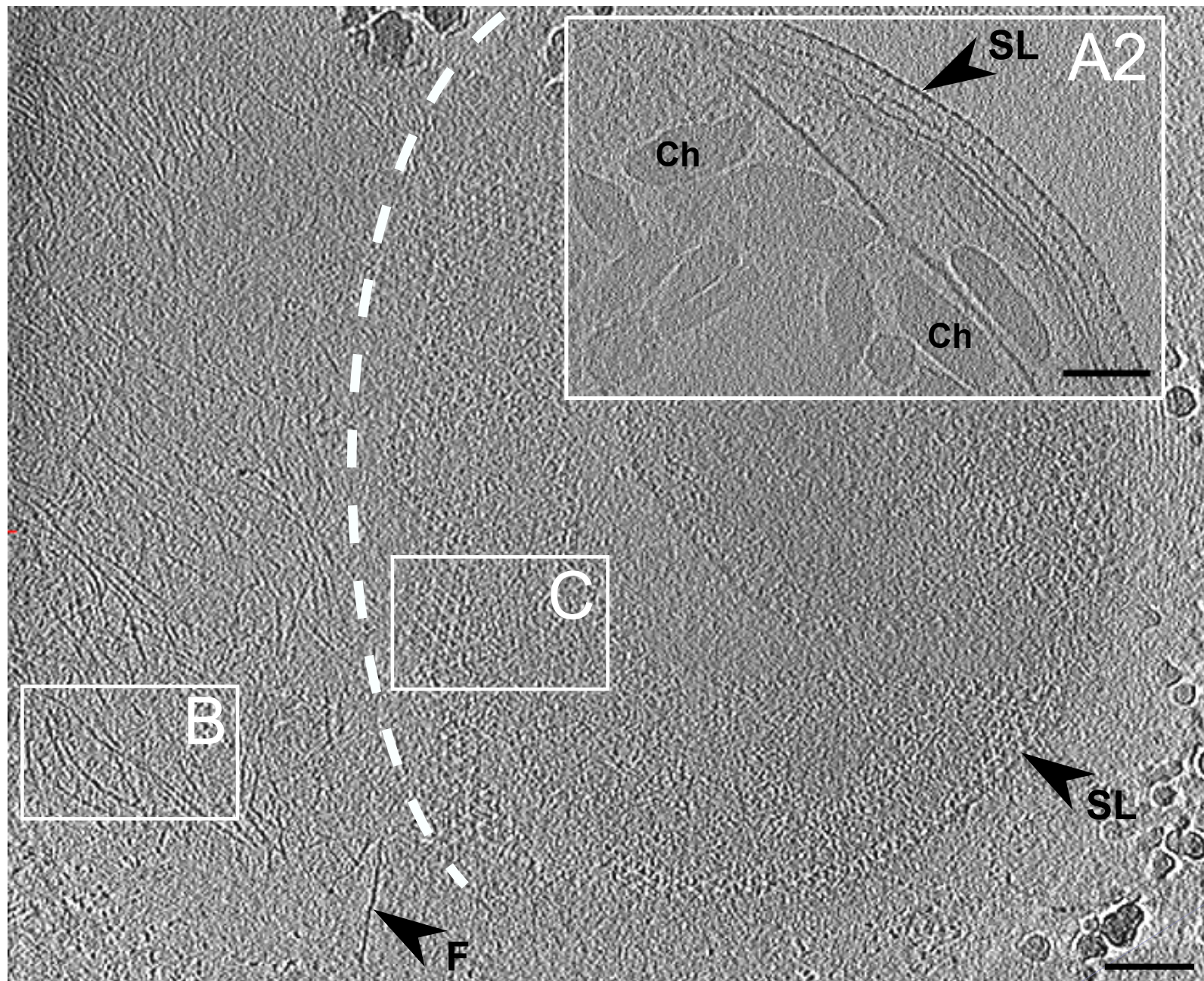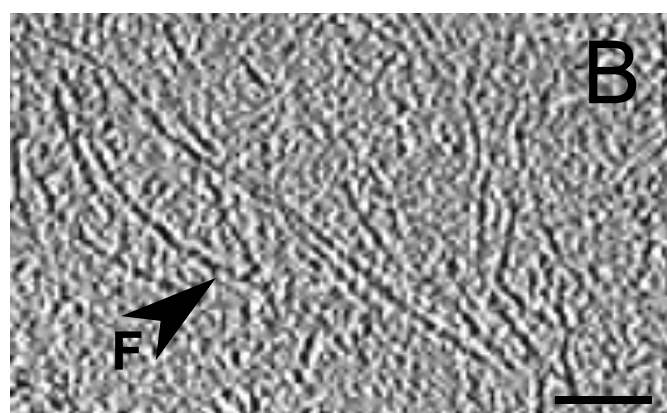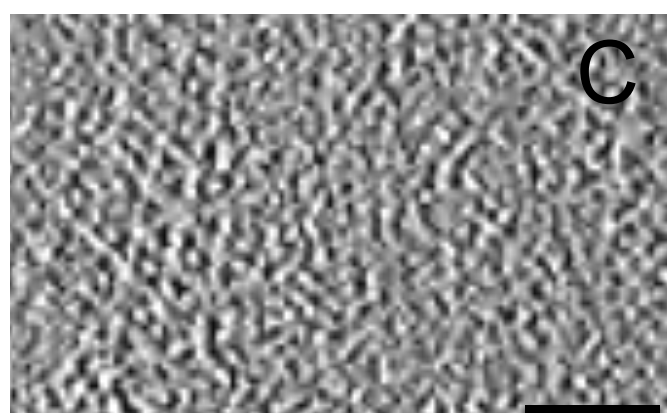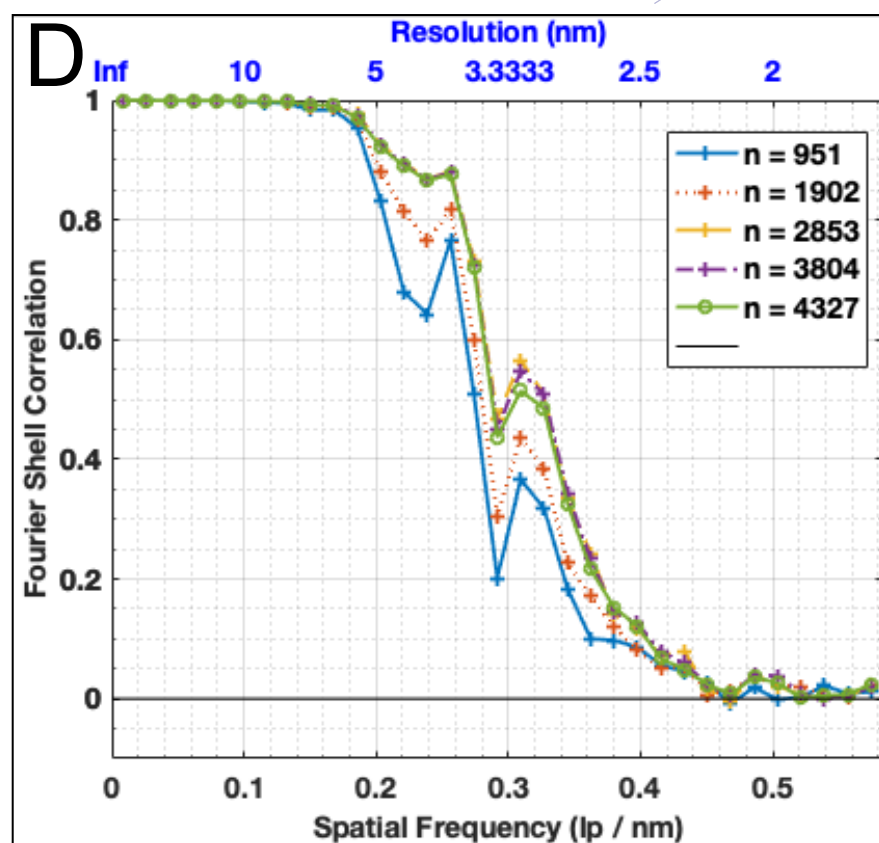

Supplement: Supplementary file 3 [file Data_Sheet_1.zip › Data Sheet 1.pdf]

# Supplementary Figure S2

A *R. castenholzii*

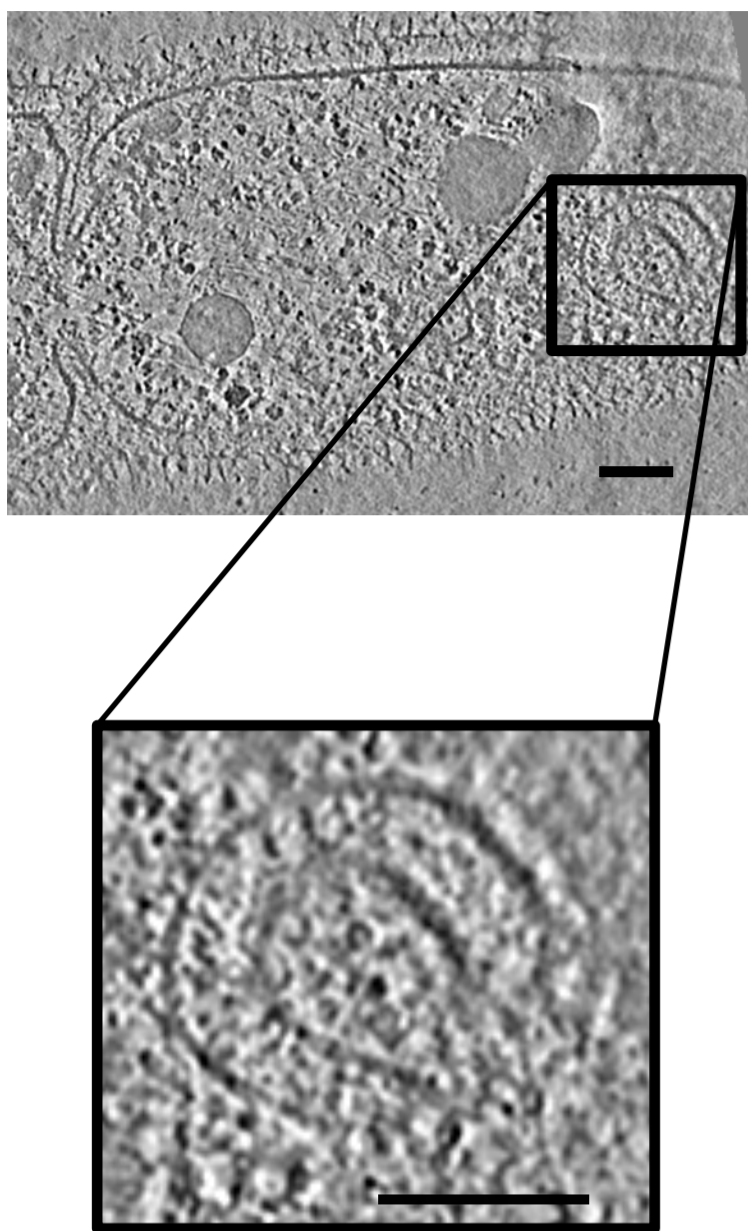

*C. aggregans*

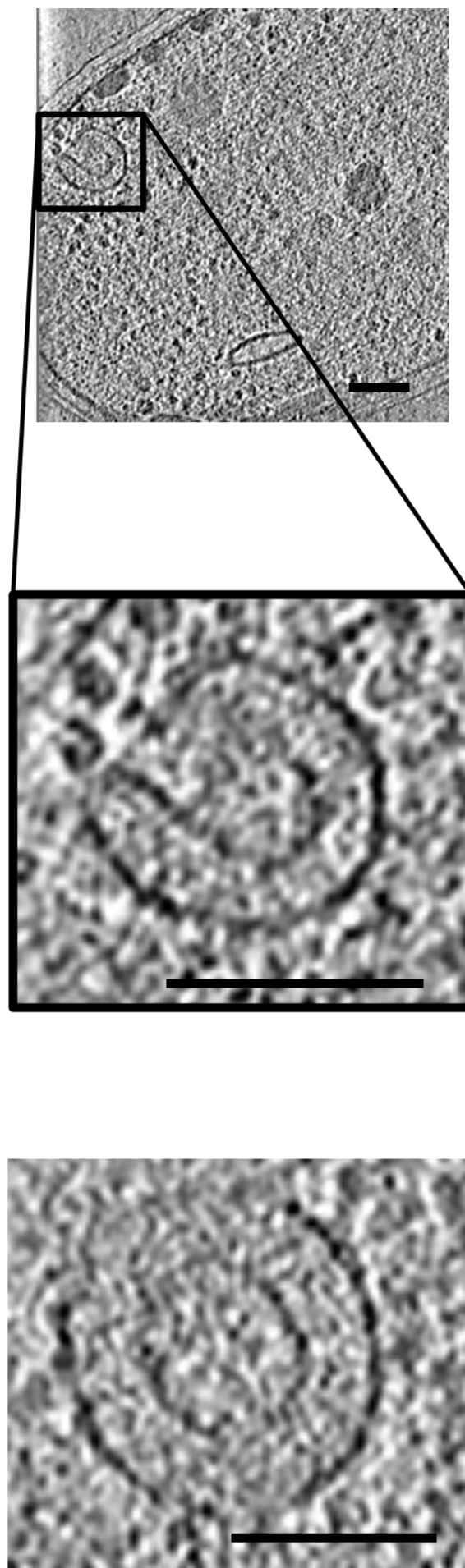

'*Ca. Viridilinea mediisalina*'

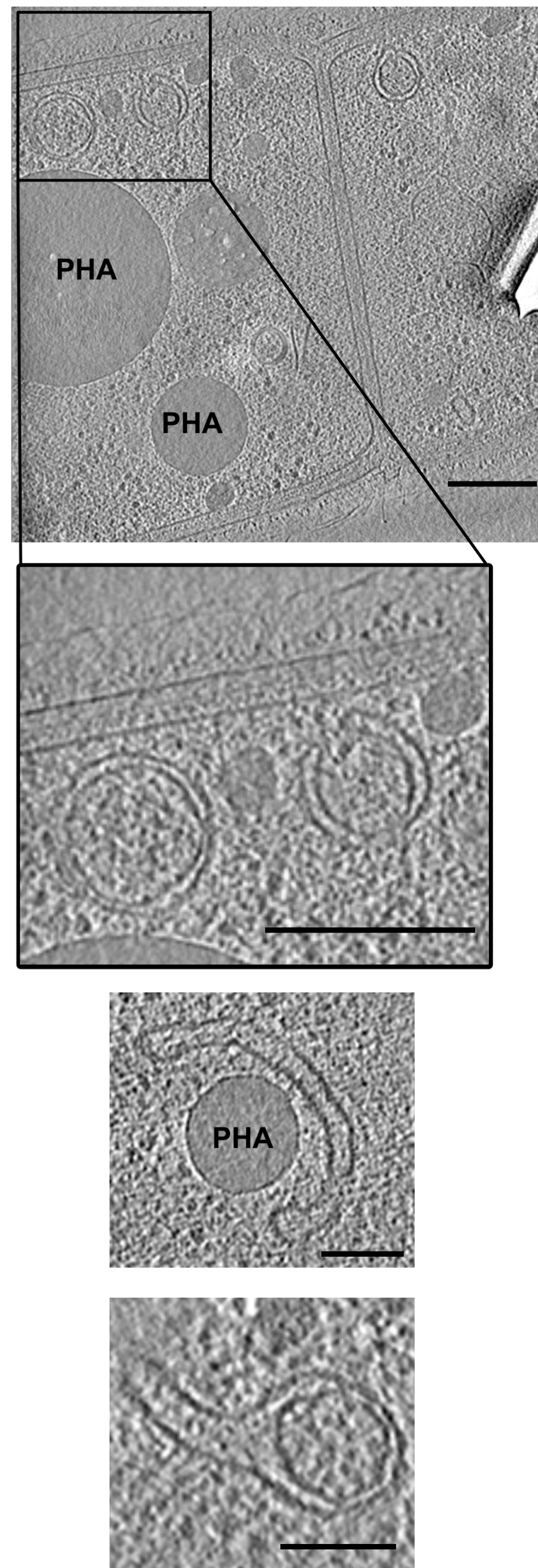

B

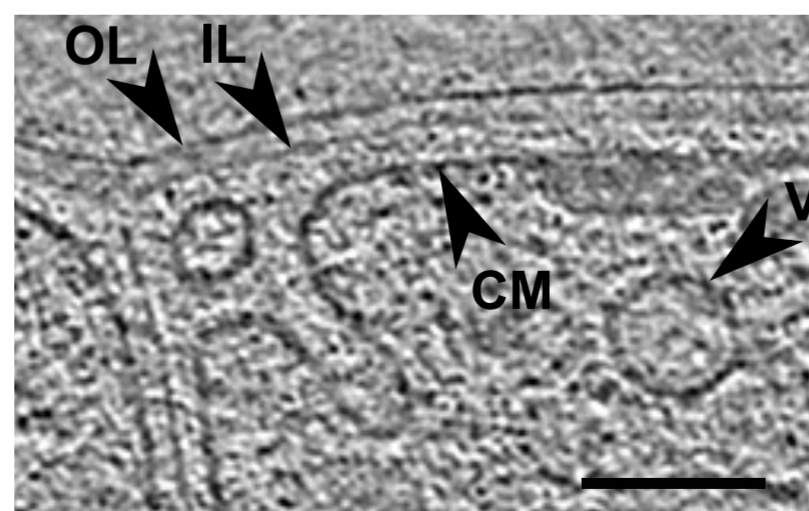

Supplement: Supplementary file 3 [file Data_Sheet_1.zip › Data Sheet 2.pdf]
